# Landscapes of gut microbiome and bile acid signatures and their interaction in HBV-associated acute-on-chronic liver failure

**DOI:** 10.3389/fmicb.2023.1185993

**Published:** 2023-05-18

**Authors:** Zhiwei Bao, Runan Wei, Xiaoping Zheng, Ting Zhang, Yunjiao Bi, Sijia Shen, Pengfei Zou, Junjie Zhang, Huadong Yan, Ming D. Li, Zhongli Yang, Hainv Gao

**Affiliations:** ^1^State Key Laboratory for Diagnosis and Treatment of Infectious Diseases, National Clinical Research Center for Infectious Diseases, National Medical Center for Infectious Diseases, Collaborative Innovation Center for Diagnosis and Treatment of Infectious Diseases, The First Affiliated Hospital, Zhejiang University School of Medicine, Hangzhou, China; ^2^Department of Infectious Diseases, ShuLan (Hangzhou) Hospital Affiliated to Zhejiang Shuren University Shulan International Medical College, Hangzhou, China; ^3^The Second Clinical Medical College of Zhejiang Chinese Medical University, Hangzhou, China; ^4^Research Center for Air Pollution and Health, Zhejiang University, Hangzhou, China

**Keywords:** acute-on-chronic liver failure, submassive hepatic necrosis, acute decompensation, bile acids (BAs), gut microbiome

## Abstract

**Introduction:**

Submassive hepatic necrosis (SMHN, defined as necrosis of 15–90% of the entire liver on explant) is a likely characteristic pathological feature of ACLF in patients with hepatitis B cirrhosis. We aimed to comprehensively explore microbiome and bile acids patterns across enterhepatic circulation and build well-performing machine learning models to predict SMHN status.

**Methods:**

Based on the presence or absence of SMHN, 17 patients with HBV-related end-stage liver disease who received liver transplantation were eligible for inclusion. Serum, portal venous blood, and stool samples were collected for comparing differences of BA spectra and gut microbiome and their interactions. We adopted the random forest algorithm with recursive feature elimination (RF-RFE) to predict SMHN status.

**Results:**

By comparing total BA spectrum between SMHN (−) and SMHN (+) patients, significant changes were detected only in fecal (*P* = 0.015). Compared with the SMHN (+) group, the SMHN (−) group showed that UDCA, 7-KLCA, 3-DHCA, 7-KDCA, ISOLCA and α-MCA in feces, r-MCA, 7-KLCA and 7-KDCA in serum, γ-MCA and 7-KLCA in portal vein were enriched, and TUDCA in feces was depleted. PCoA analysis showed significantly distinct overall microbial composition in two groups (*P* = 0.026). Co-abundance analysis showed that bacterial species formed strong and broad relationships with BAs. Among them, *Parabacteroides distasonis* had the highest node degree. We further identified a combinatorial marker panel with a high AUC of 0.92.

**Discussion:**

Our study demonstrated the changes and interactions of intestinal microbiome and BAs during enterohepatic circulation in ACLF patients with SMHN. In addition, we identified a combinatorial marker panel as non-invasive biomarkers to distinguish the SMHN status with high AUC.

## Introduction

Although the definition of acute-on-chronic liver failure (ACLF) remains controversial, it is recognized as the most serious complication of acute decompensated (AD) cirrhosis with a 28-day mortality of 40–50% when it occurs (Li et al., 2016). Deranged systemic inflammatory responses are one of the core clinical elements of ACLF (Laleman et al., 2011; Olson and Kamath, 2011). Although AD itself has high mortality rates, the pro-gression to ACLF increases this mortality dramatically^4^; ACLF is the main cause of death in decompensated cirrhosis^5^. Even after active treatment, some patients will develop ACLF on the basis of AD, while some patients will not. The underlying pathogenesis is still unclear. From AD to ACLF, the differences in intestinal flora between the two groups of patients are worth further exploration.

Several studies have reported specific microbiome patterns in the stool of patients with ACLF (Bajaj et al., 2014; Zhang et al., 2019). The potential pathogens of *Pasteurellaceae, Streptococcaceae*, and *Enterococcaceae* were more abundant in the ACLF group than in the health control (HC) group, and the relative abundance of *Bacteroidaceae, Ruminococcaceae*, and *Lachnospiraceae* was decreased (Chen et al., 2015). In addition, *Lachnospiraceae* might be a biomarker of ACLF, whereas the relative abundance of *Pasteurellaceae* could predict the mortality rate (Chen et al., 2015). Bile acids (BAs) are the end products of hepatic cholesterol metabolism, synthesized in the liver, and transported across the canalicular membrane of hepatocytes as primary conjugated BAs into the biliary system, which ultimately drains them into the small intestine. Within the intestine, gut microbiota modulates the biological activity of BAs through enzymatic modifications, producing secondary BAs. Recent data highlighted the role of BAs as essential mediators of the gut–liver crosstalk (Wahlstrom et al., 2016; Schneider et al., 2018). For instance, BAs regulate specific host metabolic pathways and modulate inflammatory responses via G protein-coupled and nuclear receptors, such as the G protein-coupled BA receptor 1 (TGR5) and farnesoid X-activated receptor (FXR), respectively (Aguiar Vallim et al., 2013; Chiang, 2013). However, the differences in the microenvironment in gut and BA profiles between ACLF and AD patients are largely unknown. Is it possible that the difference in intestinal microflora between patients with the two diseases leads to a different prognosis for the disease?

A recent prospective cohort study reported that submassive hepatic necrosis (SMHN) is a likely characteristic pathological feature of ACLF in patients with hepatitis B cirrhosis (Li et al., 2015). Compared with AD patients who did not have SMHN, patients with SMHN had a higher level of anti-inflammatory cytokines, as shown by serum cytokine, gene expression, and immunohistochemical studies (Li et al., 2015). However, these reports did not investigate intestinal microecology and bile acid metabolism as well as their relationship on the basis of pathology.

In this study, we collect serum, portal venous blood, and stool and liver tissue samples from patients with end-stage liver disease on the day of transplantation. BA spectrum analysis, metagenomics sequencing, and histopathological typing were performed to fully elucidate the changes in bile acid composition in decompensated cirrhosis with different pathological conditions (with or without SMHN) and the interaction between BA and gut microbiota across enterohepatic circulation.

## Methods

### Participants

This was a cross-sectional study conducted at a Liver Transplantation Center at the Shulan (Hangzhou) Hospital, Zhejiang Shuren University School of Medicine from 2018 to 2019. All patients or their representatives provided written informed consent to participate in this study. A total of 33 patients with chronic liver disease for an AD on the hospitalized waiting list were included in this study. AD was defined as described previously (Li et al., 2015). Since this study was focused on patients with chronic HBV infection, 16 patients were excluded either because they had another single etiology (e.g., alcohol, drug, and autoimmune) or because they were co-infected with HCV or human immunodeficiency virus. Clinical/biochemical characteristics were collected systematically during hospitalization. In addition, two common scoring systems [Child–Pugh (Pugh et al., 1973) and MELD (Kamath et al., 2007)] associated with the clinical prognosis were calculated. This study was approved by the Ethics Committee of Shulan (Hangzhou) Hospital, Zhejiang Shuren University School of Medicine.

### Histology

After total hepatectomy for subsequent LT, two wax specimens of the left liver and right liver and one specimen of the caudate lobe were collected from each patient for histological examination. SMHN was defined as necrosis of 15–90% of the entire liver on explant. The presence of SMHN was determined according to the Iskak score (Ishak et al., 1995). After histological evaluation by two experienced pathologists, patients were divided into two groups, with or without SMHN.

### Statistical analysis

Statistical analysis was performed using R software (R Foundation for Statistical Computing, v. 4.0.3; https://www.r-project.org/). The group differences of baseline information in the training and validation cohorts were assessed using the chi-square test or the Fisher exact test for categorical variables and the *t*-test or the Mann–Whitney *U*-test for continuous variables, as appropriate. The Kruskal–Wallis test was used for comparing more than two groups. Multivariate hypothesis testing for BAs profile and microbiome composition data was carried out via permutational analysis of variance (PERMANOVA) for comparisons between the SMHN (+) and SMHN(-) groups. *P*-values of < 0.05 were considered significant. ^*^P < 0.05, ^**^P < 0.01, and ^***^P < 0.001.

## Results

### Clinical characteristics of the patients

A total of 17 patients comprising ACLF (*n* = 6) and AD (*n* = 11) were eligible for inclusion (Supplementary Figure S1). All ACLF patients were diagnosed as SMHN (+) and further confirmed by histology. In total, two out of 11 clinical decompensation cirrhosis patients were diagnosed as SMHN (+), whereas nine patients were not diagnosed as SMHN (+). Thus, eight patients were included in the SMHN (+) group. Nine out of 11 patients were enrolled in the SMHN(-) group. Two patients in the SMHN(-) group had concurrent small hepatocellular carcinoma, which met the Milan criteria.

As shown in Table 1, the median ages of SMHN (+) and SMHN(-) patients were 41.8 ± 12.4 and 52.8 ± 8.9 years, and 94% were men. Compared with the SMHN(-) group, patients with SMHN (+) had more advanced liver disease, higher frequencies of ascites and HE, more marked impairment of liver function tests, and higher Child-Pugh scores and model for end-stage liver disease (MELD) scores.

**Table 1:** The clinical and demographic characteristics of the sample.

	**SMHN + (N=8)**	**SMHN - (N=9)**	***P*-value**
**Demographic**			
Age	41.8 ± 12.4	52.8 ± 8.9	
Male sex	8 (100%)	8 (89%)	
**Clinical**			
**Identifible precipitating events**	7 (87.5%)	3 (33.3%)	
Hastrointestinal bleeding	1 (12.5%)	2 (22.2%)	
Infection	2 (25%)	1 (11.1%)	
Hepatitis flare	4 (50%)	0 (0%)	
**Biochemical parameters**			
Tbil	425.0 (38.5, 487)	34.1 (24.0, 91.0)	0.019
Albumin	29.3 (28.1, 30.1)	29.6 (26.7, 30.8)	0.455
Cr	65.5 (60.8, 88.3)	70.0 (59.0, 79.0)	0.4
PT	27.0 (21.8, 35.4)	18.3 (15.7, 21.8)	0.074
INR	2.3 (1.9, 3.0)	1.6 (1.4, 1.9)	0.073
Cholesterol	4.9 (4.2, 6.5)	3.6 (2.9, 3.9)	0.047
TBA	265.5 (216.5, 301.7)	191.3 (69.1, 258.9)	0.723
**Hepatic encephalopathy**	4 (50%)	1 (11.1%)	
**Refractory ascites**	8 (100%)	4 (44.4%)	
**Using antibiotic**	6 (75%)	1 (11.1%)	
**Child-Pugh score**	11.0 (8.8, 12.0)	7.0 (6.0, 10.0)	0.066
**MELD score**	17.9 (15.1, 20.7)	11.7 (8.9, 15.5)	0.03

### Comparison of multi-omic profile differences between the SMHN(-) and SMHN(+) groups

**Figure 1:**
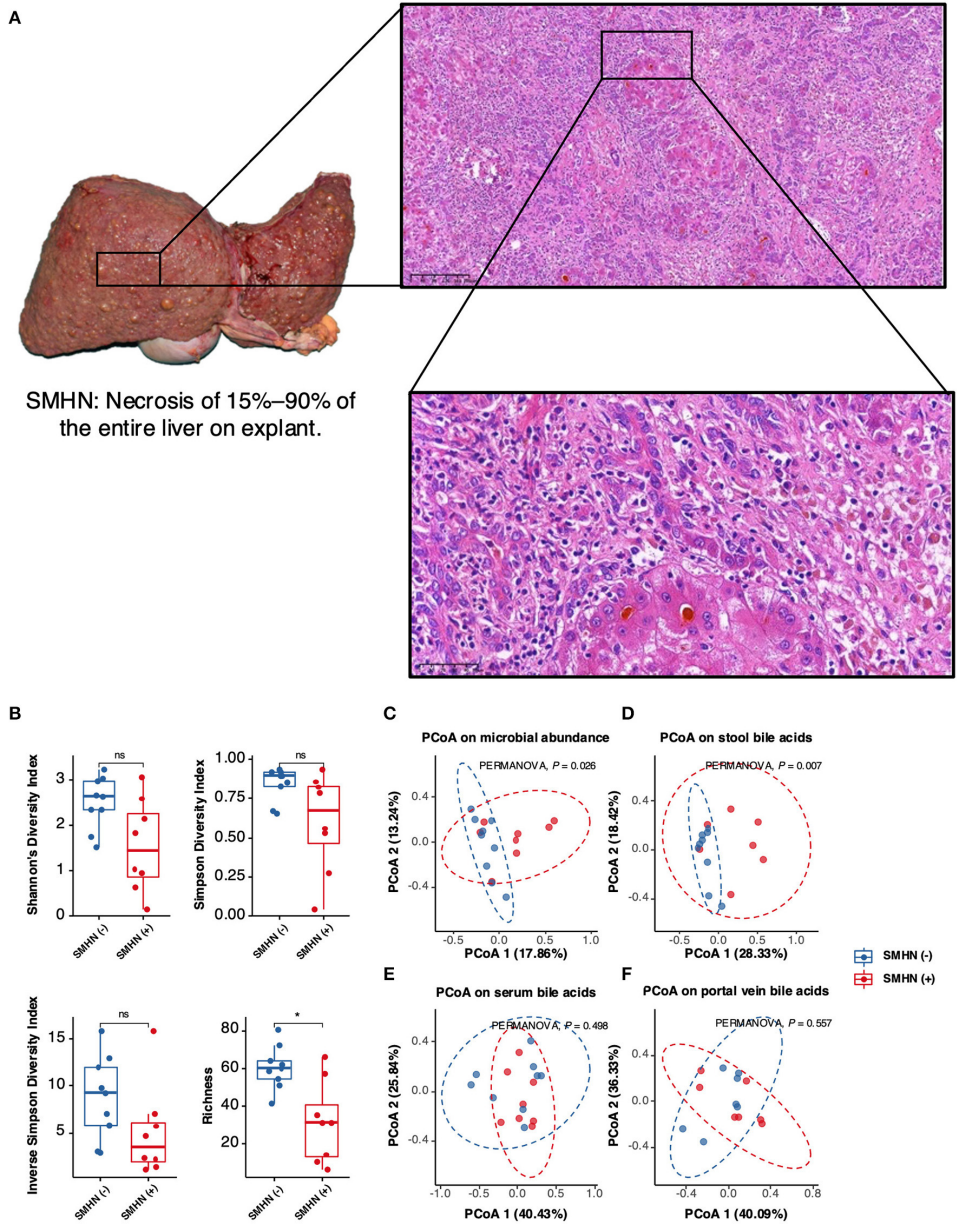
Multi-omic characteristics in SMHN(-) versus SMHN (+). **(A)** Alpha diversity in SMHN(-) and SMHN (+) patients. ^*^P < 0.05. **(B–E)** Principal coordinates analysis (PCoA) based on bacterial signatures, stool BAs, serum BAs, and portal vein BAs (Bray–Curtis distance).

The Shannon diversity index, Simpson diversity index, and inverse Simpson diversity index revealed a significant difference between the two groups, with greater bacterial alpha diversity detected in the SMHN(-) patients (Figure 1A). PCoA analysis reviewing that bacterial signatures between the two groups were significantly different (PERMANOVA, *P* = 0.026; Figure 1B).

**Figure 2:**
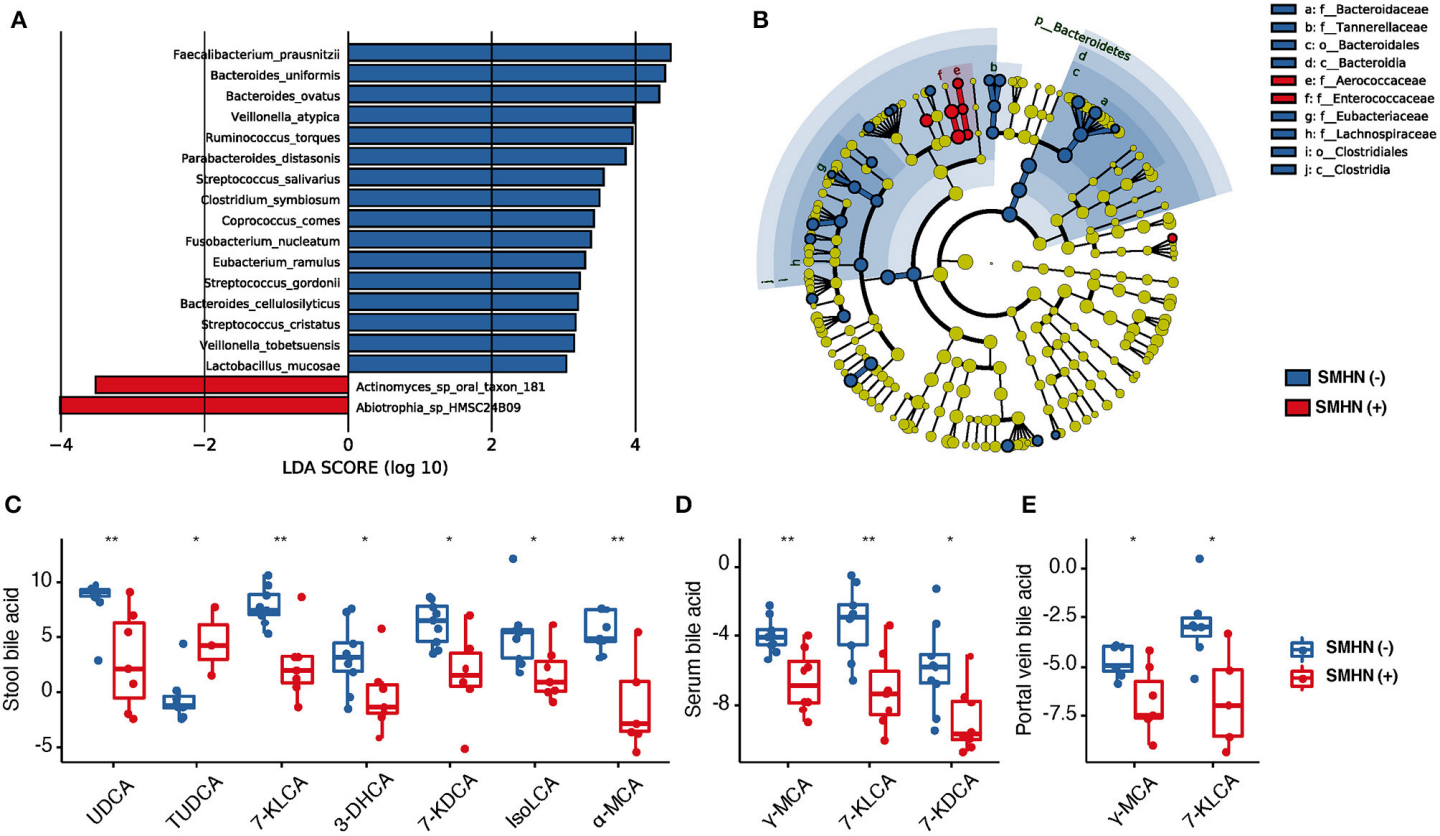
The bacterial species, portal vein BAs, serum BAs, and fecal BAs that discriminate SMHN(-) from SMHN (+). **(A)** LDA analysis showed significant bacteria species differences between SMHN(-) and SMHN (+). **(B)** A cladogram of different taxonomic compositions in SMHN(-) and SMHN (+). **(C–E)** Significant different BAs in stool, serum, and portal vein. *P < 0.05; **P < 0.01.

Together, we identified 18 discriminative bacterial species between the SMHN(-) and SMHN(+) groups, with the LEfSe algorithm (*P* < 0.05; Figure 2A; Supplementary Table S1). Compared with the subjects in the SMHN(+) group, SMHN(-) patients were characterized by 16 enriched species mainly belonging to the orders of Bacteroidales, Clostridiales, and Lactobacillales and two depleted species (*Abiotrophia_sp_HMSC24B09, Actinomyces_sp_oral_taxon_181*). To further explore these findings, we additionally included healthy group samples for multi-class comparisons. *Abiotrophia_sp_HMSC24B09* and *Actinomyces_sp_oral_taxon_181* remained significantly enriched in the SMHN(+) group, while *Ruminococcus_torques, Eubacterium_ramulus, and Coprococcus_comes* were significantly decreased compared with subjects in the SMHN(-) group and the healthy control group (Supplementary Table S2). LDA analysis at all taxonomic levels further showed that the SMHN(-) subjects were enriched by two classes, two orders, and four families, whereas the SMHN (+) subjects were only enriched by two families (*Aerococcaceae* and *Enterococcaceae*) (Figure 2B; Supplementary Table S3).

The overall fecal bile acid signatures of the SMHN(-) group were significantly different from that in the SMHN(+) group (PERMANOVA, *P* = 0.007; Figure 1C). However, no significant changes were detected in serum BAs and portal vein BAs (Figures 1D, E). Based on the Spearman correlation analysis of BAs from serum, porta vein, and stool, we found that there existed significant positive correlations among them (i.e., serum vs. portal vein: *R* = 0.95, *P* = 2.2e-16; portal vein vs. stool: *R* = 0.22, *P* = 4.5e-05; and serum vs. stool: *R* = 0.22, *P* = 6.7e-05).

Compared with the SMHN(+) group, the SMHN(-) group displayed significant enrichments in six stool BAs (i.e., UDCA, 7-KLCA, 3-DHCA, 7- KDCA, IsoLCA, and α-MCA), three serum BAs (r-MCA, 7-KLCA, and 7-KDCA), two portal vein BAs (i.e., γ-MCA and 7-KLCA), and depletion in TUDCA in stool samples (Figures 2C–E).

Compared with the healthy controls, we found that most of the detected significant BAs (i.e., UDCA, γ-MCA, 7-KLCA, 7-KDCA, and α-MCA) were higher in the SMHN(-) patients but lower in the SMHN (+) patients, although they all showed a similar pattern. There were also some bile acids that showed a trend of increasing (i.e., TUDCA) or decreasing (i.e., AlloCA) gradients from healthy controls to SMHN(-) and SMHN (+) patients (Supplementary Figure S2). Significant differences in the total fecal BA concentrations were detected between healthy controls and SMHN(-) or SMHN(+) patients as well as between SMHN(-) and SMHN(+) patients (Supplementary Figure S3). Furthermore, we found a significantly higher concentration of the secondary bile acids and bile acid metabolites in the SMHN(-) stool samples compared to the SMHN(+) group and a similar pattern for the primary BAs in the stool (Wilcoxon, *P* = 0.07; Supplementary Figure S4), indicating that intestinal microbiome mediates the disorder of BAs in SMHN(+) patients.

We, next, explored the direct roles of the gut microbiome in modulating BA metabolism. According to the previous report, *Clostridium* and *Eubacterium* contain an enzyme for 7α-dehydroxylation (Jia et al., 2018), which impacts the concentrations of 7-KLCA and 7-KDCA in the intestine. These BAs can return to the liver through the portal vein and then finally enter into the systemic circulation (Supplementary Figure S5).

### Co-abundance analysis among the bacteria and BAs

**Figure 3:**
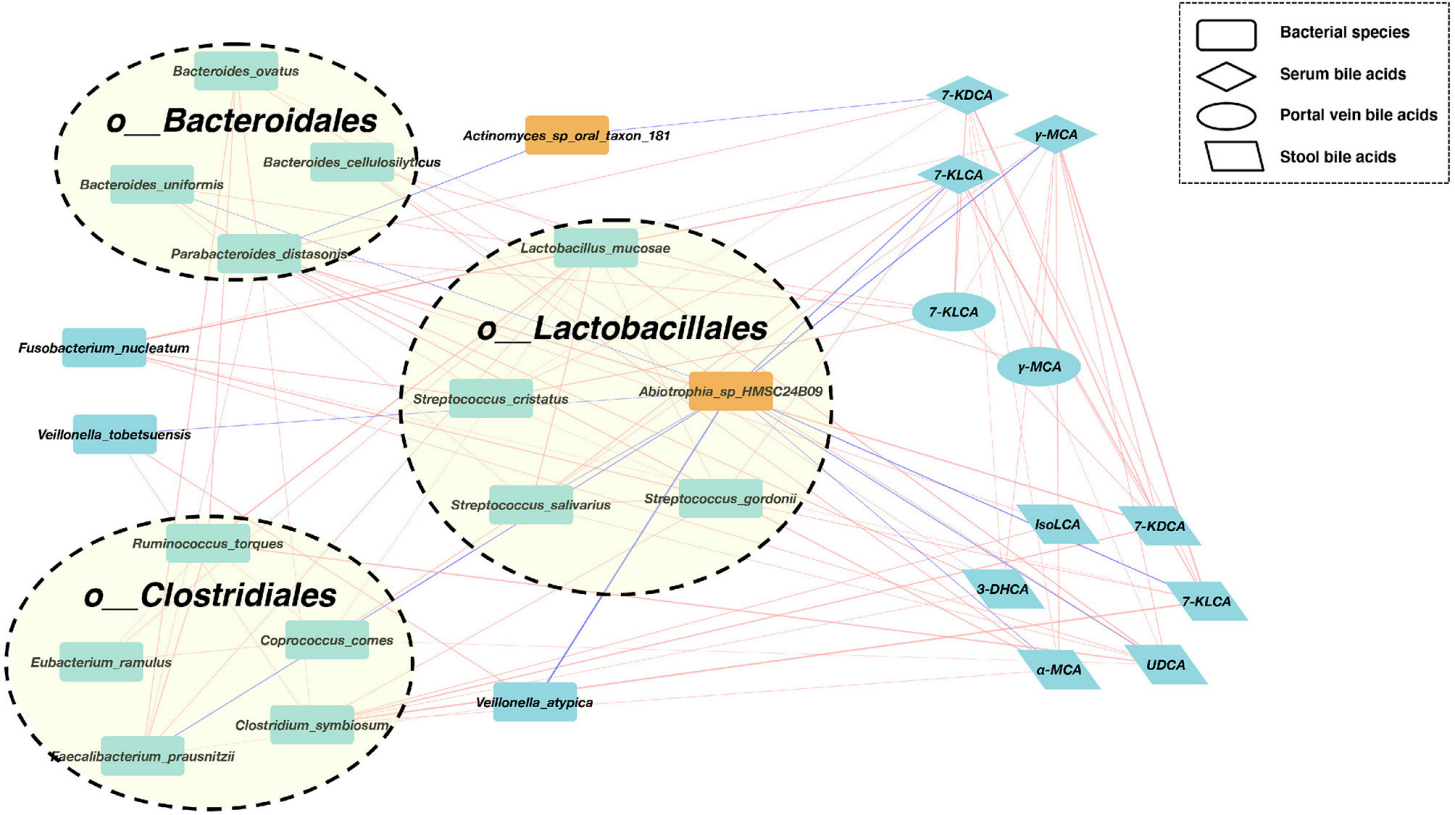
A co-abundance network constructed from the relative abundances of differential bacterial species, portal vein BAs, serum Bas, and fecal BAs in SMHN(-) vs. SMHN (+). Correlation networks in patients reaching outcomes filtered at *P* < 0.05 between microbiota (orange nodes) and metabolites (cyan-blue nodes). Red edges indicate negative while blue indicates positive correlation.

We, next, explored the correlations of abundances for these differential gut bacterial species and BAs in enterohepatic circulation (Figure 3). In the SMHN(-) group, compared to the SMHN (+) group, we observed four enriched bacterial species from the order Bacteroidales, five enriched bacterial species from the order Clostridiales, four enriched bacterial species from the order Lactobacillales, and one depleted species from the order Lactobacillales.

We further found that *Abiotrophia*_sp_HMSC24B09 enriched in the SMHN (+) group exhibited negative correlations with four bacterial species (*Bacteroides uniformis, Streptococcus salivarius, Faecalibacterium prausnitzii*, and *Veillonella atypica*) and five BAs (7-KLCA and γ-MCA in serum, 7-KLCA, UDCA, and α-MCA in stool). Another depleted species in the SMHN(-) group was *Actinomyces_sp_oral_taxon_181*, which was negatively correlated with *Parabacteroides distasonis* and 7-KDCA in serum.

To identify key regulators involved in modulating the bacterial and BA processes, we calculated all node degrees of the differential bacteria–BA network. Defining a hub as having a node degree of >5, we identified 21 hub nodes, including 12 species, five stool BAs, three serum BAs, and one portal vein BA (Supplementary Table S4). Notably, *P. distasonis*, a member of the Bacteroidales order, exhibited the highest node degree of 12, with positive correlations with five bacteria species (i.e., *Bacteroides ovatus, Bacteroides uniformis, Faecalibacterium prausnitzii, Clostridium symbiosum*, and *Streptococcus cristatus*), 7-KDCA in serum and portal vein, and four stool BAs (IsoLCA, 3-DHCA, α-MCA, and 7-KDCA). Among BAs, 7-KLCA and γ-MCA in serum had the highest node degree. Serum 7-KDCA showed positive correlations with four species (*Fusobacterium nucleatum, Streptococcus cristatus, Streptococcus salivarius*, and *Streptococcus gordonii*), two portal vein BAs (7-KLCA and γ-MCA), and three stool BAs (7-KLCA, 7-KDCA, and UDCA). The γ-MCA in serum showed positive correlations with two species (*Fusobacterium nucleatum* and *Streptococcus salivarius*), two portal vein BAs (7-KLCA, γ-MCA), and five stool BAs (7-KLCA, 7-KDCA, UDCA, α-MCA, and 3-DHCA).

### Association of microbial function alterations with SMHN status

**Figure 4:**
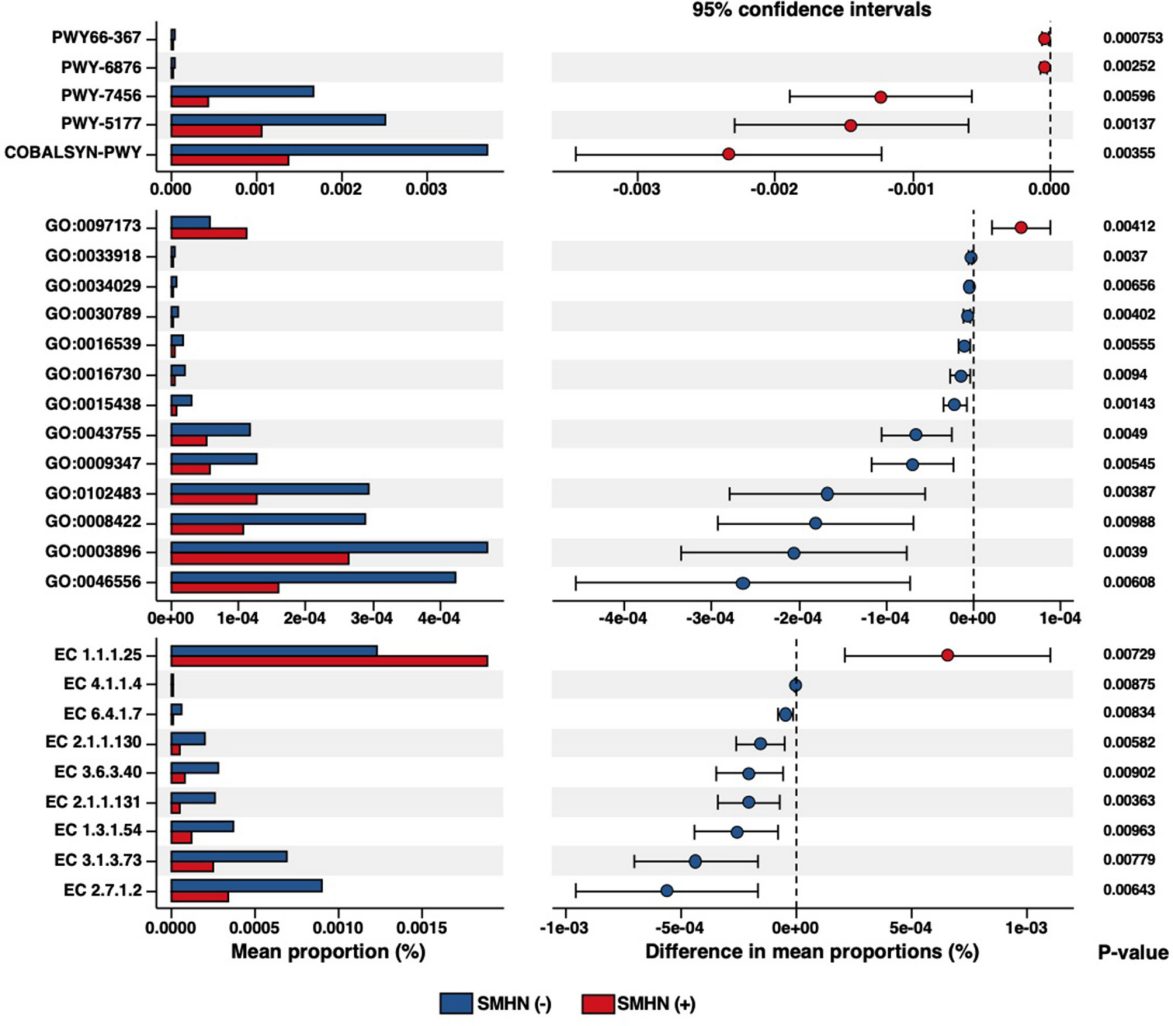
Differential biological pathways, GO, and enzymes in SMHN(-) or SMHN (+) subjects.

To further explore the SMHN status associated with functional dysbiosis, we performed functional profiling, which revealed a total of five differential Metacyc pathways, 13 differential GO terms, nine differential enzymes, seven differential KO identifiers, 120 differential eggnog accessions, and 37 differential Pfam accessions (Figure 4, Supplementary Tables S5–S7).

The differential MetaCyc pathways were mainly involved in Ketogenesis (PWY66-367), isopropanol biosynthesis (PWY-6876), β-(1, 4)-mannan degradation (PWY-7456), glutaryl-CoA degradation (PWY-5177), and adenosylcobalamin salvage from cobinamide I (COBALSYN-PWY). Compared with SMHN (+) patients, SMHN(-) patients were characterized by 10 enriched GO molecular function terms. The N-acetylmuramic acid catabolic process (GO: 0097173) displayed enrichment in SMHN (+) patients, and intein-mediated protein splicing (GO: 0016539) displayed enrichment in SMHN(-) patients.

### Combinatorial biomarkers for discriminating SMHN(-) from SMHN (+)

**Figure 5:**
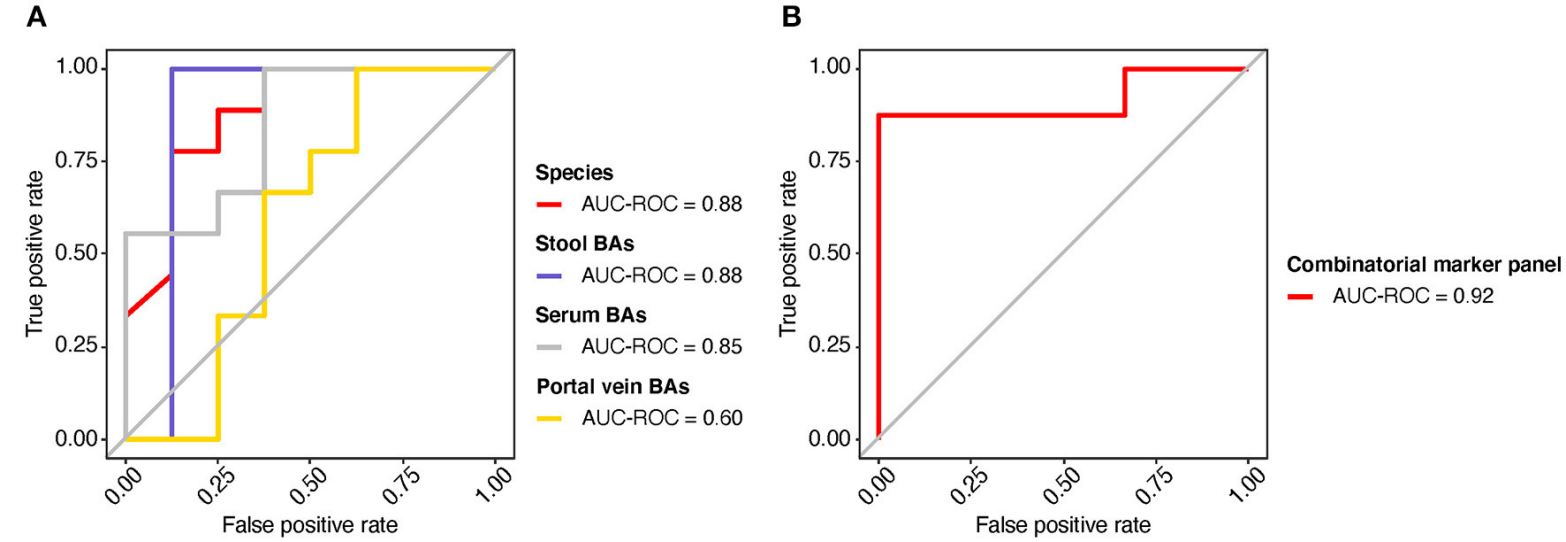
Predicting the SMHN status from multi-omic features. **(A)** ROC curves for the models based on bacterial species, stool BAs, serum BAs, and portal vein BAs. **(B)** ROC curve for the combinatorial marker panel.

The potential value of gut metagenomic and BA markers was further investigated in SMHN (+) diagnosis using machine learning models based on multi-omics data. A recursive feature elimination procedure with a random forest algorithm was used to identify the representative features (Supplementary Figures S9, S10). The random forest algorithm was also used to develop prediction models. We found that individual marker panels could discriminate patients with SMHN(-) from SMHN (+) subjects with an area under the curve (AUC) ranging from 0.60 to 0.88 (bacterial species: AUC = 0.88; Stool BAs: AUC = 0.88; Serum BAs: AUC = 0.85; and Portal vein BAs: AUC = 0.60; Figure 5A).

Among the bacterial characteristics selected in the model, 11 bacterial species overlapped with the results of the LDA analysis (Supplementary Figure S9). Furthermore, we found that a combinatorial marker panel of multi-omic biomarkers enabled discriminating SMHN(-) from SMHN (+) subjects with a high classification power (AUC = 0.92, Figure 5B). The markers used in the combinatorial model include nine bacterial species, 15 stool BAs, five serum BAs, and one portal vein BAs (Supplementary Figure S11).

## Discussion

In this study, we presented the landscapes and interaction networks of differential gut bacterial species, stool bile acids, serum bile acids, and portal vein bile acids in decompensated cirrhosis patients with different pathological conditions (with or without SMHN). We identified a combinatorial marker panel that can distinguish the SMHN status with a high AUC. These findings lay the foundation for understanding the roles of the overall gut–liver ecosystem in submassive hepatic necrosis, which further supports the notion that ACLF is a separate entity from liver cirrhosis with a distinct pathophysiology.

Previous reports have commonly focused on using plasma metabolite signatures, potentially deriving from gut microbiota, to differentiate ACLF from AD (Claria et al., 2016, 2019; Horvatits et al., 2017; Moreau et al., 2020). These studies clearly demonstrate that there is a bioenergetics failure coupled with a systemic inflammatory response in ACLF patients compared to those with decompensated cirrhosis. In the current study, since we performed bile acid quantification on serum, portal vein blood, and stool samples, it allowed us to study the flow of bile acids in the hepatoenteric circulation much more straightforwardly and explore the BA correlations in different sampling sites. We found there were significant differences in stool bile acid composition between the two groups. Gut microbes are involved in the metabolic process of secondary bile acids and play a key role in submassive hepatic necrosis. For example, UDCA can be produced by bacterial epimerization of the primary bile acid chenodeoxycholic acid and signifies the presence of specific beneficial *Clostridium* and *Ruminococcus* species (Winston and Theriot, 2020). Such species can also form 7-KLCA or 7-KDCA and secondary bile acids, and their relative reduction likely represents a bacterial functional alteration that predisposes patients to mortality and ACLF (Doden et al., 2018). These bile acids that differ in the intestine will further flow back to the liver through the portal vein, where BA-mediated effects may impact disease progression. Previous studies have found that BAs seem to directly activate inflammatory pathways in hepatocytes during cholestasis by stimulating the production of proinflammatory mediators (Gujral et al., 2004; Allen et al., 2011). Furthermore, BA signaling via FXR and TGR5 activation may attenuate inflammatory damage in the liver at a lower concentration (Xu et al., 2012; Shaik et al., 2015).

It is now widely accepted that gut bacteria can significantly shape several metabolic pathways in the host. Previous studies mainly characterized the gut microbiome in patients with cirrhosis using 16S rRNA gene amplicon sequencing technology with a phylogenetic resolution at the genus level or the family level (Bajaj et al., 2014, 2017, 2018, 2019; Chen et al., 2015). Consistent with our findings, some bacterial species from Bacteroides, Clostridiales, and Lactobacillales are the main bacterial orders of the gut microbiota involved in BA metabolism (Kullak-Ublick et al., 2004; Sayin et al., 2013). Moreover, *P. distasonis* (belonging to order Bacteroides), the species with the highest node degree in our differential species–BA network, can generate the succinate and secondary bile acids and plays key roles in the modulation of host metabolism. It has been reported that treatment with live *P. distasonis* can alter the bile acid profiles of mice, increasing the levels of LCA and UDCA, and in turn, reducing hyperlipidemia by activating the FXR pathway, and as a result, repairing gut barrier integrity (Wang et al., 2019). Previous studies have found that after ceftriaxone treatment, UB. ceftriaxensis and *P. distasonis* were driving two observed monodominance community states that peaked at 92 and 95% relative abundance, respectively. *P. distasonis*, the main driver of the second postantibiotic response stage, has been associated with faster microbiome recovery after antibiotic interventions^50^. The predominance of *P. distasonis* means the production of more secondary bile acids and the re-establishment of intestinal homeostasis. The difference in *P. distasonis* between ACLF and AD patients might be the main reason for the different progress of the two diseases. These findings could form the basis of future clinical trials focusing on changing the bile acids or the intestinal flora, such as the use of prebiotics, probiotics, and even fecal transplantation in hospitalized patients with liver cirrhosis. In addition, *Bacteroides ovatus* and *Bacteroides uniformis* (both belonging to order Bacteroides) were found to present at higher levels in healthy controls than in ulcerative colitis (UC) or irritable bowel syndrome (IBS) patients, suggesting a possible protective role played by this group of bacteria (Noor et al., 2010; Lopez-Siles et al., 2017). Overall, the disruption of bile acid metabolism caused by intestinal microbes may affect not only gut microbiota but also the whole circulatory system of patients.

However, there were some limitations regarding this study. First, the sample size of our cohort was relatively small, and thus, our developed model requires further validation in independent samples. Second, although we first detected disturbances of the overall gut–liver ecology implicated in submassive hepatic necrosis and provided a comprehensive and multilevel understanding of the role of the disturbed gut microbiome, we did not investigate the specific mechanisms underlying such a disturbance using animal models. Third, how intestinal flora and metabolites induce SMHN in HBV-related cirrhotic livers was not answered by our data.

To the best of our knowledge, this is the first study to comprehensively explore microbiome and BA patterns across the enterohepatic circulation. Especially, the characteristics of bile acids from the portal vein in patients with or without SMHN. For the first time, our study revealed that *P. distasonis* may play a role in the prognosis of ACLF and AD. Based on multi-omics data, using the random forest algorithm, we developed a combinatorial marker panel with a high AUC value. These findings provide new directions to uncover pathogenesis and develop novel diagnostic strategies for submassive hepatic necrosis.

## Data availability statement

The original contributions presented in the study are included in the article/Supplementary material, further inquiries can be directed to the corresponding authors.

## Ethics statement

Written informed consent was obtained from the individual(s) for the publication of any potentially identifiable images or data included in this article.

## Author contributions

XZ and TZ conducted the pathological experiments. RW participated in tissue and clinical data collection. ZB and ZY participated in data analysis. HG, ML, RW, ZB, and ZY participated in manuscript writing and editing. YZ and HG conceived the study and were involved in every step of this study. All authors approved the manuscript as submitted.
