# Supplementary material for: Landscapes of gut microbiome and bile acid signatures and their interaction in HBV-associated acute-on-chronic liver failure
Source: Front Microbiol. 2023 May 18;14:1185993. doi: 10.3389/fmicb.2023.1185993 (PMC10233926; doi:10.3389/fmicb.2023.1185993)
Supplement: Supplementary file 1 [file Data_Sheet_1.zip › Suuplementary Material.docx]

**Supplementary Methods**

**Sample collection**

After collection of serum, portal vein and stool samples, they were stored at −80°C for the subsequent analysis of level of BAs. All fecal samples from patients on the day of liver transplantation were freshly collected at the hospital and frozen at −80°C until DNA extraction.

**Metagenomic analysis of fecal samples**

Fecal DNA was extracted using the AllPrep DNA/RNA Mini Kit (Qiagen). Whole-genome shotgun sequencing library was constructed with Illumina Library Preparation Kit and was sequenced on the Illumina NovaSeq 6000 (Illumina Inc., San Diego, CA, USA) platform according to the manufacturer’s recommended protocols.

The whole metagenome data were first trimmed and all reads were then filtered using KneadData with the default parameters. The relative abundance of gut microbial taxonomic units was calculated with MetaPhlan3 [1] under the default settings. Functional and pathway profiling was calculated with HUMANn3 [2] using the full-size UniRef90 database with default settings. The pathway abundance profiles were calculated by MetaCyc [3] pathway definitions and MinPath [4]. UniRef90 abundance profiles were converted to GO terms [5], KEGG modules [6], KEGG identifiers, KO identifiers, Pfam accessions [7], EC numbers [8], and eggnog accessions [9] using the “uniref90_level4ec” option of the humann_regroup_table script. Here, we annotated a total of 469 MetaCyc pathways, 5328 GO terms, 5140 KO identifiers, 7425 Pfam accessions, 2250 EC numbers and 46414 eggnog accessions.

Low-frequency (observed in <20% samples) microbial features (e.g., species, functional modules, and metabolites) were discarded. Alpha diversity analysis including Shannon, Simpson and Invsimpson indexes was conducted and visualized using the vegan package in R. Richness was calculated by number of species. Species composition data from MetaPhlan was converted to predict read counts by multiplying relative abundances with the total sequence counts. Principal coordinates analysis (PCoA) with Bray-Curtis distance was used to visually evaluate the overall difference and similarity of bacterial and bile acids between the SMHN(+) and SMHN(-) groups. The PERMANOVA was used to test group differences. The differential bacterial species between the two groups were identified using Linear discriminant analysis Effect Size (LEfSe) algorithm with p < 0.05, linear discriminant analysis (LDA) score >2.0, “all-against-all” parameter was used in multi-class analysis [10]. Moreover, Student’s t-test was used to identify the differential functional profiles for pathway abundance, GO terms, ECs, KO identifiers, eggnog accessions and Pfam accessions between the SMHN(+) and SMHN(-) groups (p < 0.01).

**Comparisons of bile acids profiles**

LC-MS (Waters ACQUITY UPLC I-Class) was used to determine the bile acid signatures of SMHN(+) and SMHN(-) subjects. The GC/MS three-dimensional matrices, including peak indexes (RT-m/z pairs), sample names (observations), and normalized peak area percentages, were imported into Multiquant software (v. 3.0.1) (SCIEX, Toronto, Canada). Log-transformed values were used in subsequent analysis.

The following BAs were assessed in this study: CA (Cholic acid), α-MCA (α-Muricholic acid), β-MCA (β-Muricholic acid), ω-MCA (ω-Muricholic acid), γ-MCA (γ-Muricholic acid), AlloCA (Allocholic acid), DCA (Deoxycholic acid), CDCA (Chenodeoxycholic acid), UDCA (Ursodeoxycholic acid), HDCA (Hyodeoxycholic acid), MoCA (Murocholic acid), TCA (Taurocholic acid), GCA (Glycocholic acid hydrate), TMCA (Tauro-α-muricholic acid), THCA (Taurohyocholic acid), GDCA (Sodium glycodeoxycholat), GCDCA (Sodium glycochenodeoxycholate), GUDCA (Glycoursodeoxycholic acid), GHDCA (Glycohyodeoxycholic acid), TDCA (Sodium taurodeoxycholate), TCDCA (Taurochenodeoxycholic acid), TUDCA (Tauroursodeoxycholic acid), THDCA (Taurohyodeoxycholic acid), LCA (Lithocholic acid), IsoLCA (Isolithocholic acid), GLCA (Glycolithocholic acid), TLCA (Taurolithocholic acid ), 3-DHCA (3-Dehydrocholic acid), 7-KDCA (7-Ketodeoxycholic acid), 7-KLCA (7-Ketolithocholic acid), 12-KLCA (12-Ketolithocholic acid), ApoCA (Apocholic acid), and 6,7-DKLCA (6, 7-Diketolithocholic acid).

Similarly, PCoA was applied to discriminate the samples from the SMHN(+) and SMHN(-) groups visually. Wilcoxon rank sum test was used to identify the differential bile acids between the SMHN (+) and SMHN (-) groups (p < 0.05).

**Combinatorial marker panel**

Feature selection was carried out by using random forest with recursive feature elimination (RF-RFE) with leave one out cross-validation and the established random forest model was then used to predict SMHN state based on the best feature subset. The receiver operating characteristic (ROC) curve was plotted to show the prediction accuracy of predicting the recurrence state. Prediction accuracy was quantified with area under the curve (AUC). The machine learning procedure was conducted by the R package caret.

**Construction of the interaction network for gut bacteria and phages**

The co-abundance networks among differential bacteria, fecal bile acids, serum bile acids and portal vein bile acids were calculated by Spearman’s rank correlation coefficient (P < 0.05). The network layout was calculated and visualized using a circular layout by the Cytoscape software [11]. The resulting network was analyzed by Cytoscape plugin CentiScaPe to calculate the topological characteristics of the network and its individual node. Key species and BAs were screened according to the degree of nodes [12].

**Legends to Supplementary Figures**

**Fig. S1.** Schematic representation of study design.

**Fig. S2.** BAs differential analysis in SMHN (-) versus SMHN (+). * BAs was significantly different between SMHN (-) patients and SMHN (+) patients (* p < 0.05, ** p < 0.01, *** p < 0.001). # BAs was significantly different in three groups (# p < 0.05, ## p < 0.01, ### p < 0.001).

**Fig. S3.** Differentially analysis of total BAs between SMHN (-) and SMHN (+). (* p < 0.05, ** p < 0.01, *** p < 0.001).

**Fig. S4.** Comparison of concentration of primary BAs, secondary BAs and BA metabolite in stool, serum, and portal vein between SMHN(-) and SMHN(+) subjects.

**Fig. S5.** Key BA metabolic pathways in gut ecosystem of SMHN (-) patients. The BAs significantly increased in SMHN (-) patients marked blue arrow.

**Fig. S6.** Predictive performance in feature selection step based on species data.

**Fig. S7.** Predictive performance in feature selection step based on stool BA, serum BAs and portal vein BAs respectively.

**Fig. S8.** The features used in combinatorial marker panel. Feature importance score was calculated by random forests algorithm.

**Reference**

1. Truong, D.T., et al., *MetaPhlAn2 for enhanced metagenomic taxonomic profiling.* Nat Methods, 2015. **12**(10): p. 902-3.

2. Abubucker, S., et al., *Metabolic reconstruction for metagenomic data and its application to the human microbiome.* PLoS Comput Biol, 2012. **8**(6): p. e1002358.

3. Caspi, R., et al., *The MetaCyc database of metabolic pathways and enzymes - a 2019 update.* Nucleic Acids Res, 2020. **48**(D1): p. D445-D453.

4. Ye, Y. and T.G. Doak, *A parsimony approach to biological pathway reconstruction/inference for genomes and metagenomes.* PLoS Comput Biol, 2009. **5**(8): p. e1000465.

5. Ashburner, M., et al., *Gene ontology: tool for the unification of biology. The Gene Ontology Consortium.* Nat Genet, 2000. **25**(1): p. 25-9.

6. Kanehisa, M., et al., *Data, information, knowledge and principle: back to metabolism in KEGG.* Nucleic Acids Res, 2014. **42**(Database issue): p. D199-205.

7. Finn, R.D., et al., *Pfam: the protein families database.* Nucleic Acids Res, 2014. **42**(Database issue): p. D222-30.

8. Bairoch, A., *The ENZYME database in 2000.* Nucleic Acids Res, 2000. **28**(1): p. 304-5.

9. Powell, S., et al., *eggNOG v4.0: nested orthology inference across 3686 organisms.* Nucleic Acids Res, 2014. **42**(Database issue): p. D231-9.

10. Segata, N., et al., *Metagenomic biomarker discovery and explanation.* Genome Biol, 2011. **12**(6): p. R60.

11. Shannon, P., et al., *Cytoscape: a software environment for integrated models of biomolecular interaction networks.* Genome Res, 2003. **13**(11): p. 2498-504.

12. Scardoni, G., M. Petterlini, and C. Laudanna, *Analyzing biological network parameters with CentiScaPe.* Bioinformatics, 2009. **25**(21): p. 2857-9.
