# Supplementary figures and images for: Landscapes of gut microbiome and bile acid signatures and their interaction in HBV-associated acute-on-chronic liver failure
Source: Front Microbiol. 2023 May 18;14:1185993. doi: 10.3389/fmicb.2023.1185993 (PMC10233926; doi:10.3389/fmicb.2023.1185993)

**Fig S1**

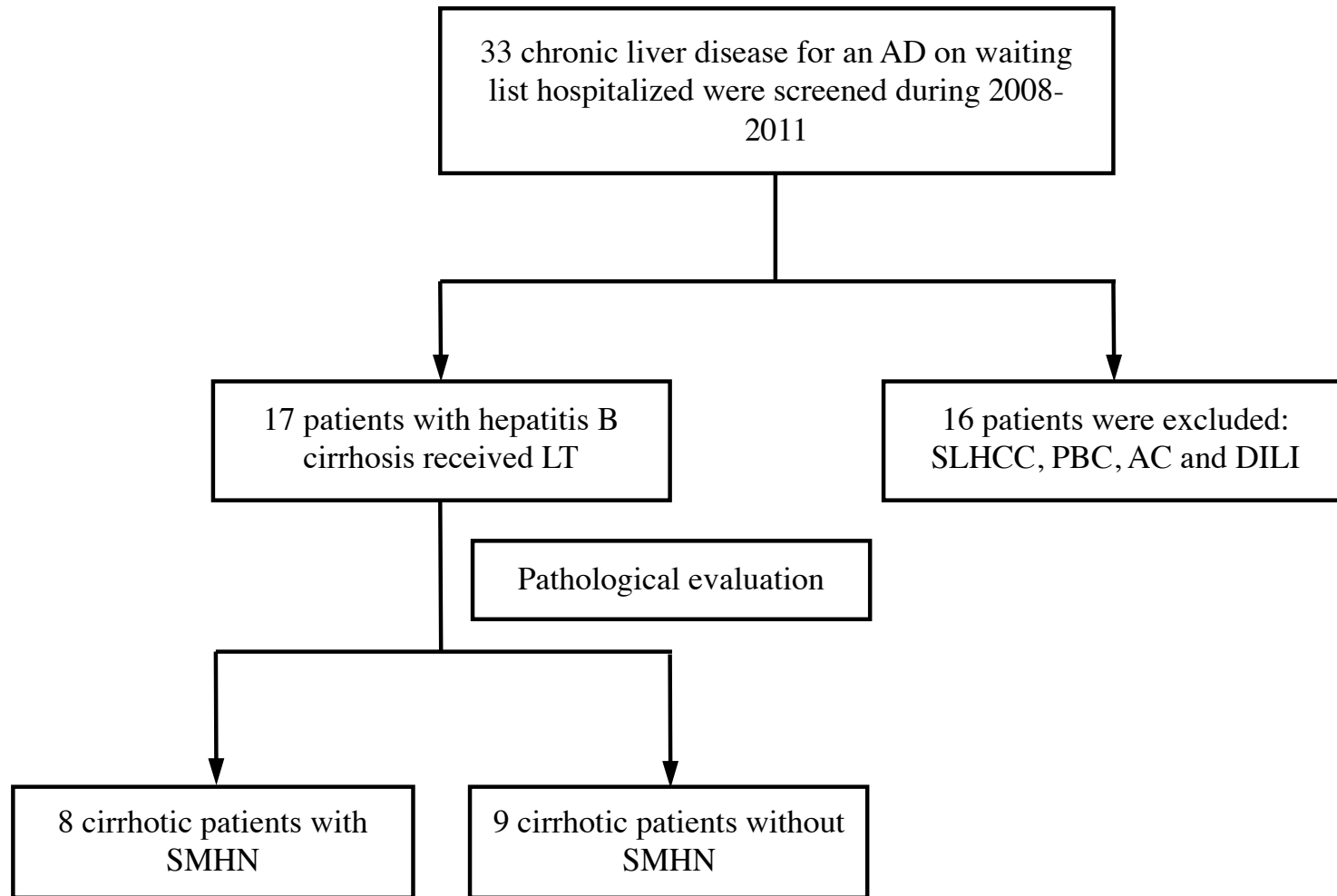

Fig S2

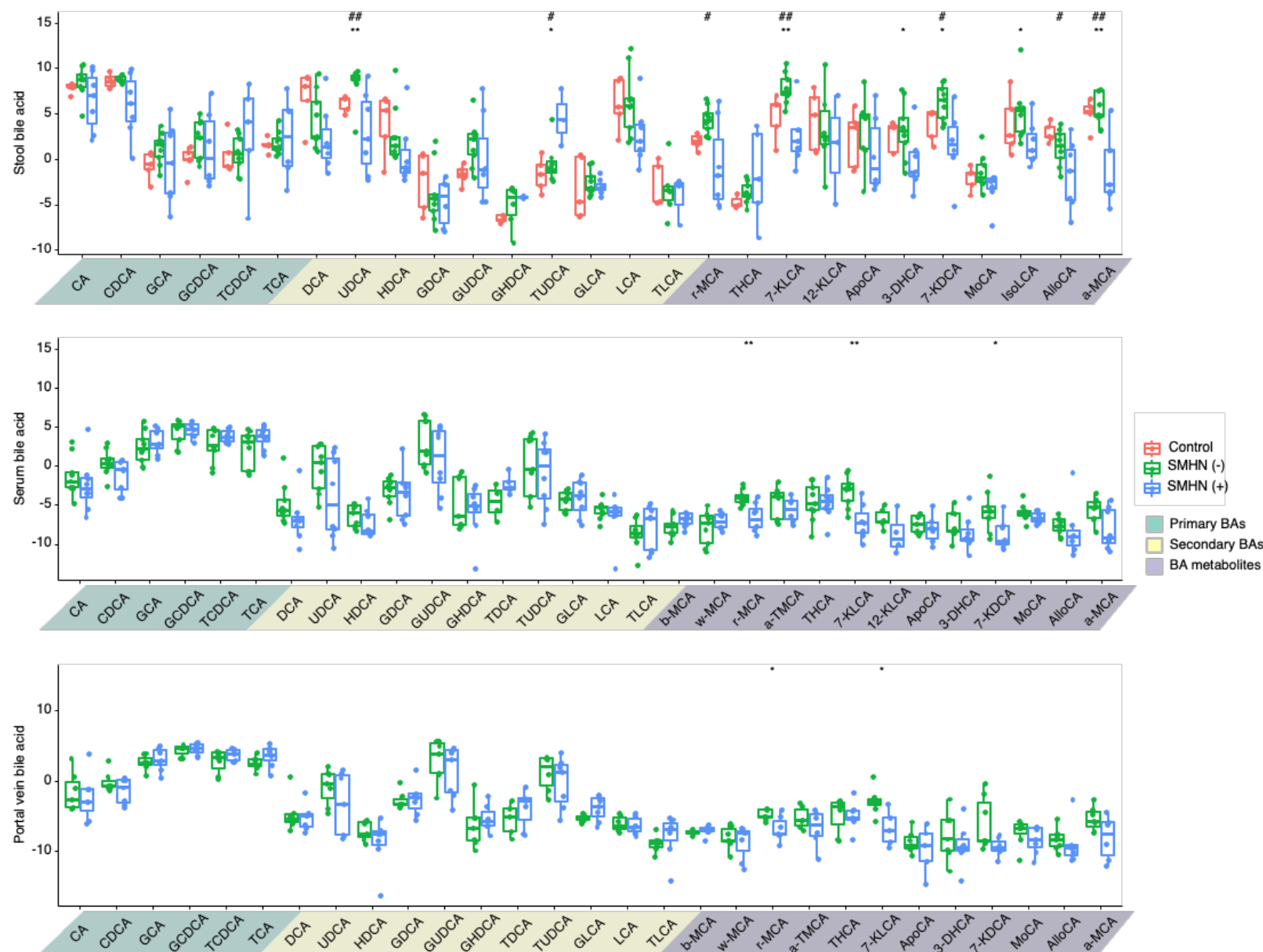

Fig S3

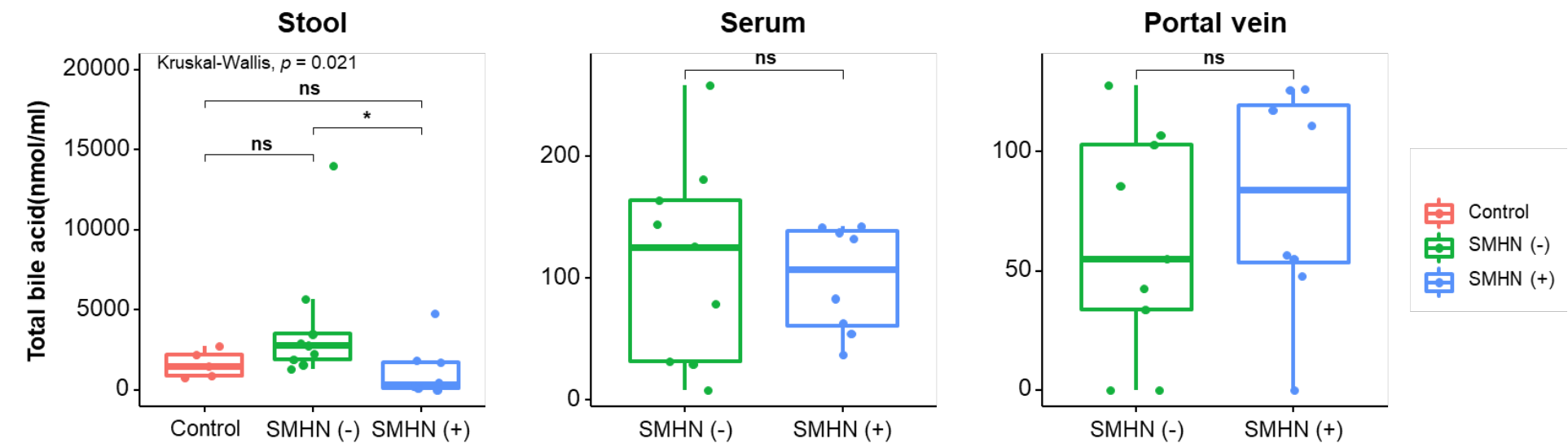

Fig S4

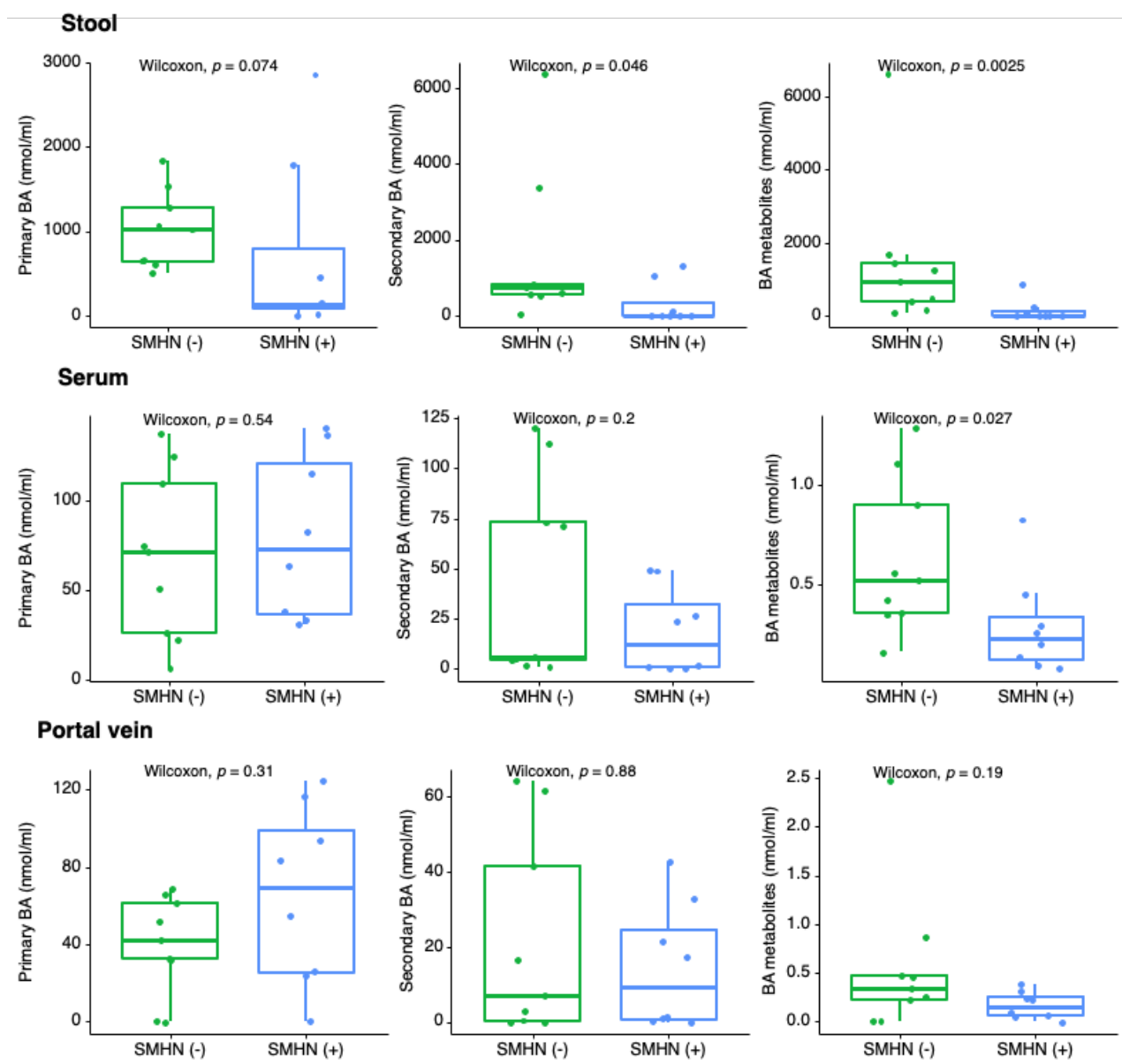

Fig S5

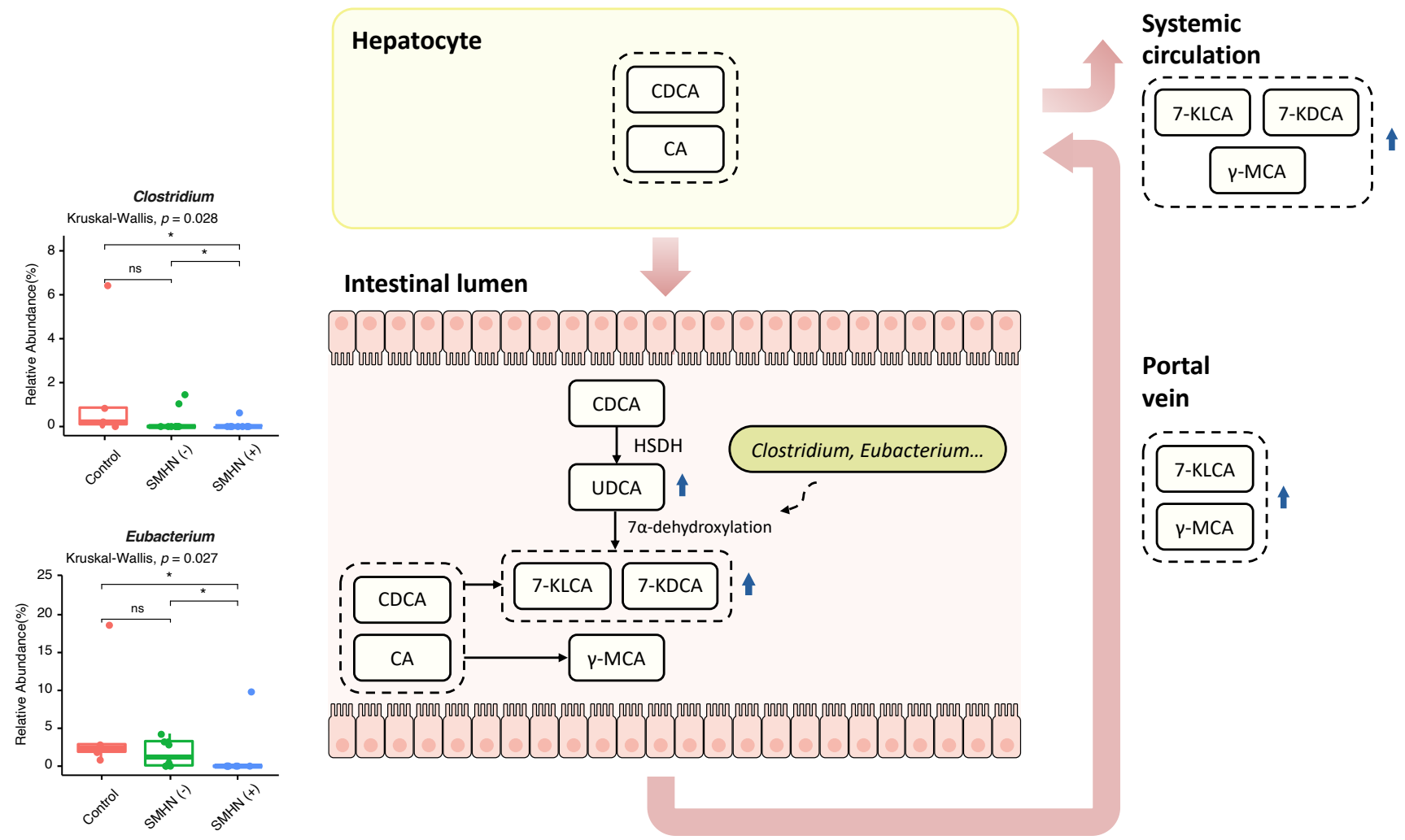

Fig S6

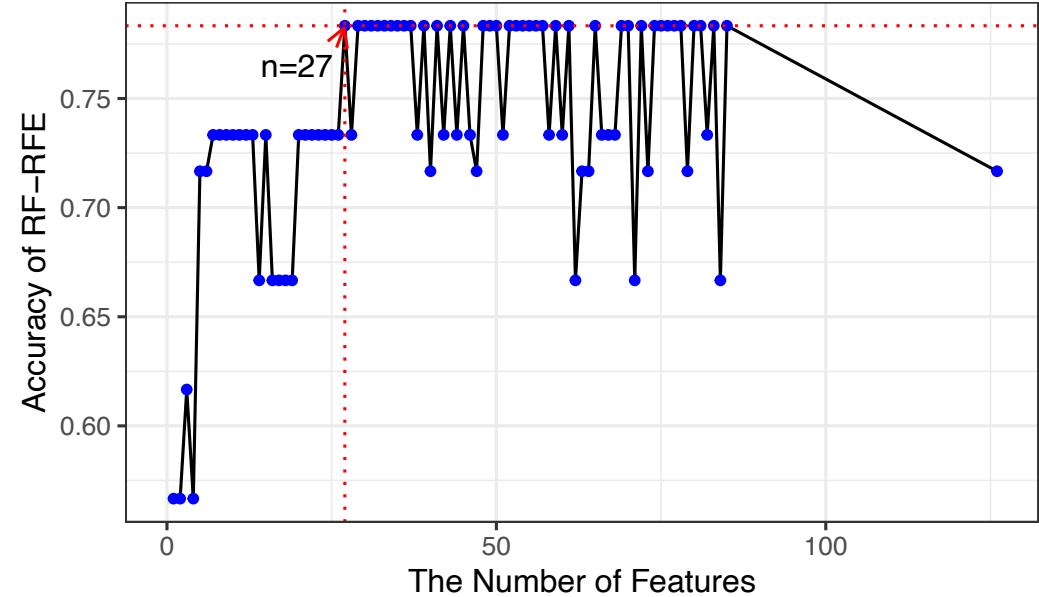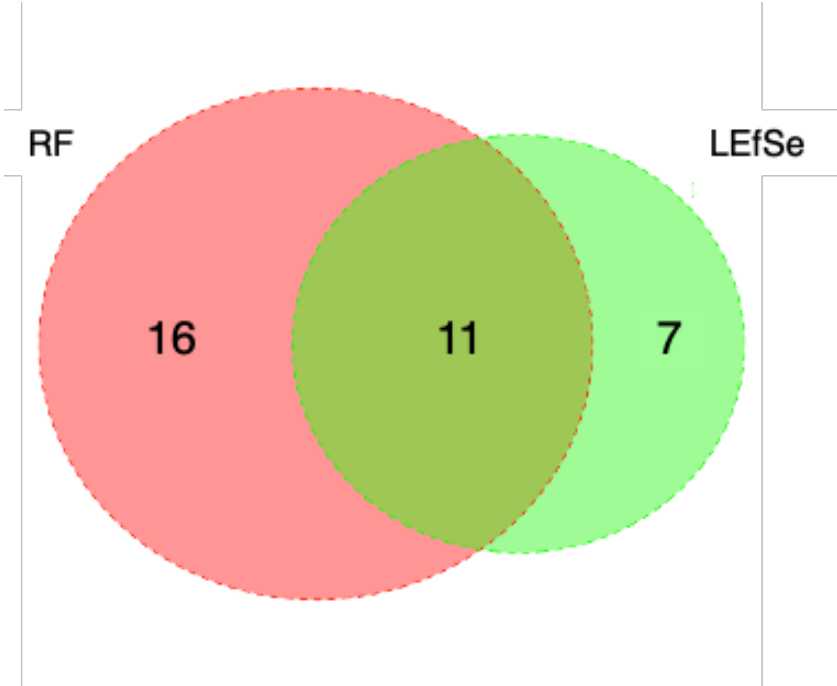

Fig S7

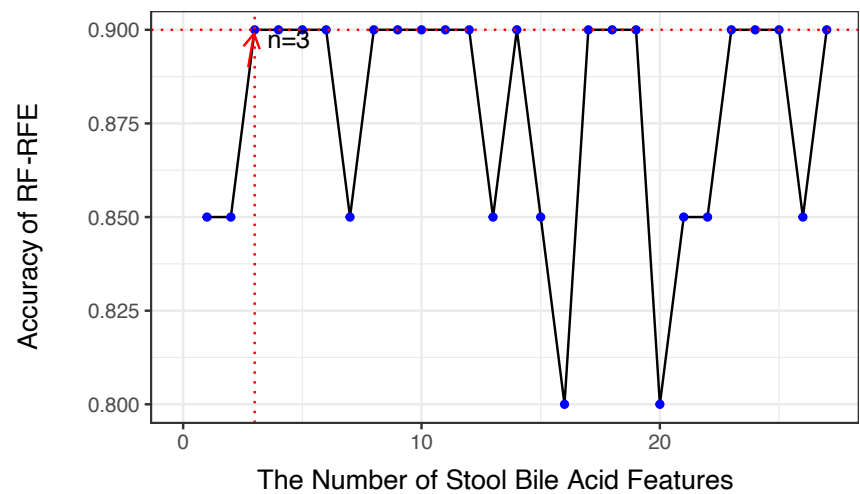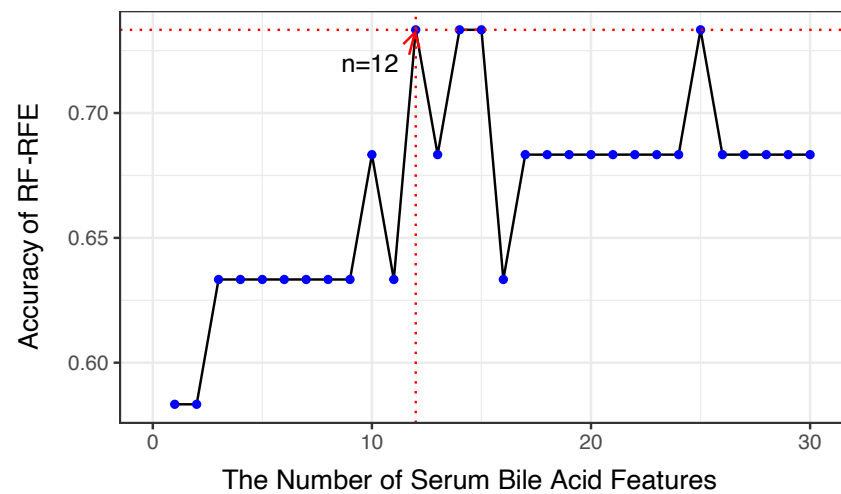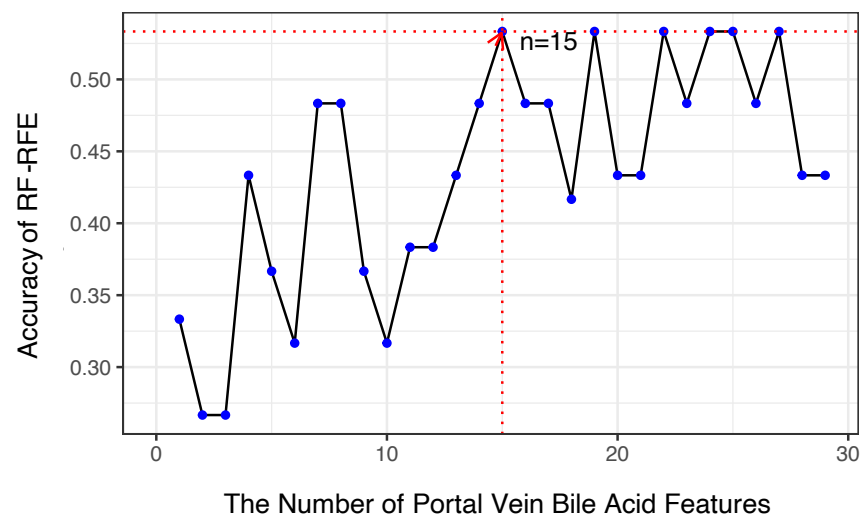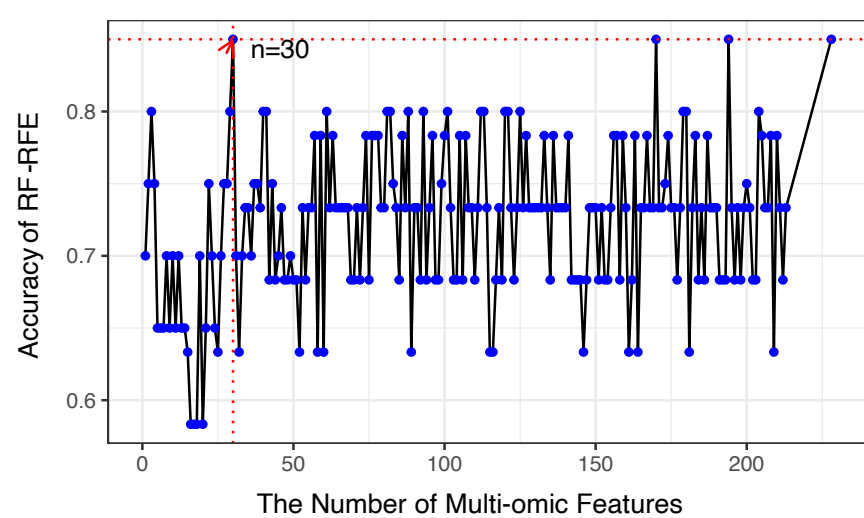

Fig S8

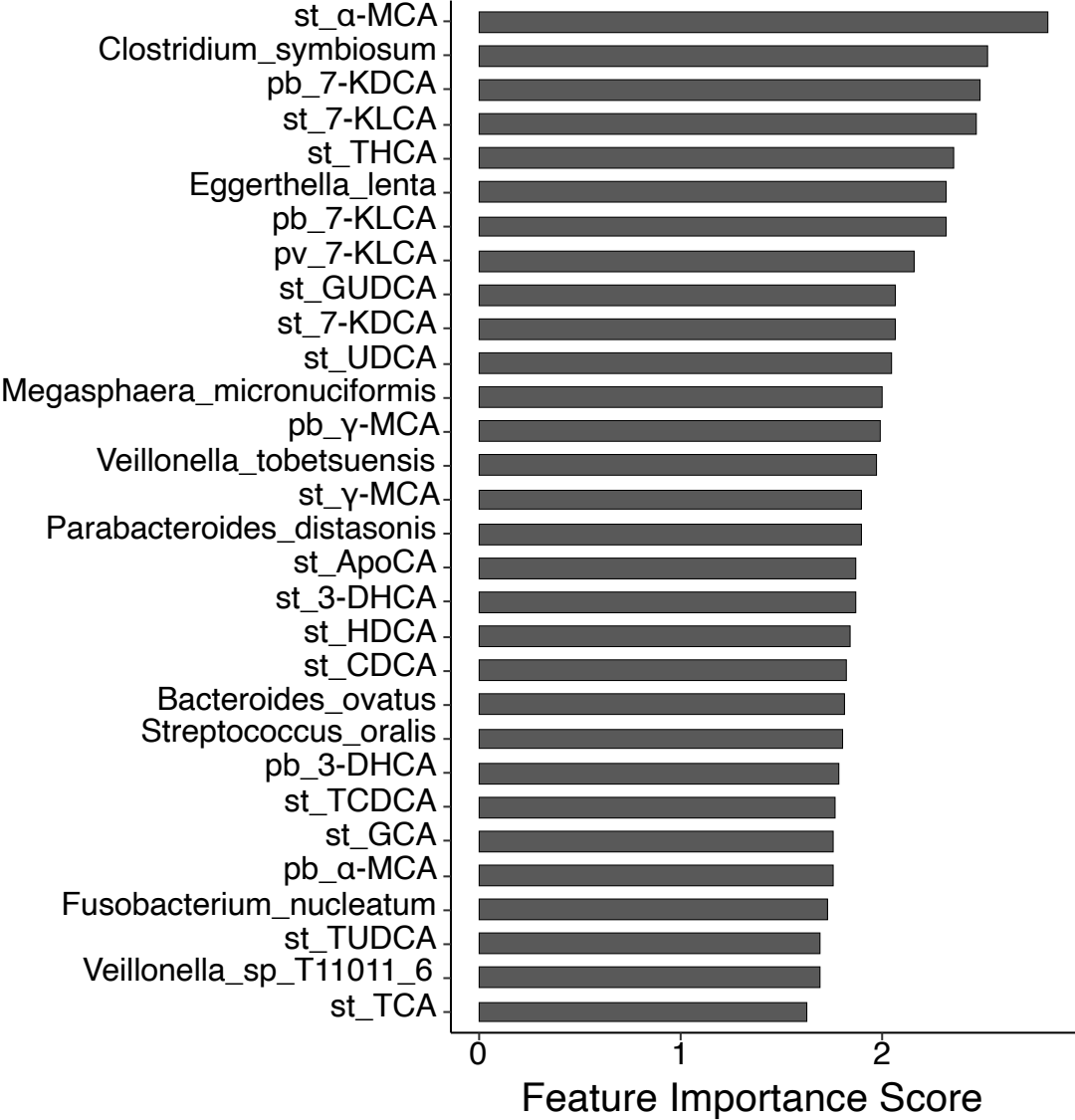

Supplement: Supplementary file 1 [file Data_Sheet_1.zip › Supplementary Figures.PDF]
